# Reproducible Machine Learning Methods for Lung Cancer Detection Using Computed Tomography Images: Algorithm Development and Validation

**DOI:** 10.2196/16709

**Published:** 2020-08-05

**Authors:** Kun-Hsing Yu, Tsung-Lu Michael Lee, Ming-Hsuan Yen, S C Kou, Bruce Rosen, Jung-Hsien Chiang, Isaac S Kohane

**Affiliations:** 1 Department of Biomedical Informatics Harvard Medical School Boston, MA United States; 2 Department of Statistics Harvard University Cambridge, MA United States; 3 Department of Pathology Brigham and Women's Hospital Boston, MA United States; 4 Department of Information Engineering Kun Shan University Tainan Taiwan; 5 Graduate Program of Multimedia Systems and Intelligent Computing National Cheng Kung University and Academia Sinica Tainan Taiwan; 6 Department of Computer Science and Information Engineering National Cheng Kung University Tainan Taiwan; 7 Department of Radiology, Athinoula A Martinos Center for Biomedical Imaging Massachusetts General Hospital Boston, MA United States; 8 Division of Health Sciences and Technology Harvard–Massachusetts Institute of Technology Boston, MA United States

**Keywords:** computed tomography, spiral, lung cancer, machine learning, early detection of cancer, reproducibility of results

## Abstract

**Background:**

Chest computed tomography (CT) is crucial for the detection of lung cancer, and many automated CT evaluation methods have been proposed. Due to the divergent software dependencies of the reported approaches, the developed methods are rarely compared or reproduced.

**Objective:**

The goal of the research was to generate reproducible machine learning modules for lung cancer detection and compare the approaches and performances of the award-winning algorithms developed in the Kaggle Data Science Bowl.

**Methods:**

We obtained the source codes of all award-winning solutions of the Kaggle Data Science Bowl Challenge, where participants developed automated CT evaluation methods to detect lung cancer (training set n=1397, public test set n=198, final test set n=506). The performance of the algorithms was evaluated by the log-loss function, and the Spearman correlation coefficient of the performance in the public and final test sets was computed.

**Results:**

Most solutions implemented distinct image preprocessing, segmentation, and classification modules. Variants of U-Net, VGGNet, and residual net were commonly used in nodule segmentation, and transfer learning was used in most of the classification algorithms. Substantial performance variations in the public and final test sets were observed (Spearman correlation coefficient = .39 among the top 10 teams). To ensure the reproducibility of results, we generated a Docker container for each of the top solutions.

**Conclusions:**

We compared the award-winning algorithms for lung cancer detection and generated reproducible Docker images for the top solutions. Although convolutional neural networks achieved decent accuracy, there is plenty of room for improvement regarding model generalizability.

## Introduction

Lung cancer is one of the most prevalent cancers worldwide, causing 1.76 million deaths per year [1,2]. Chest computed tomography (CT) scans play an essential role in the screening for [3] and diagnosis of lung cancer [4]. A randomized controlled trial demonstrated that low-dose CT screening reduced mortality from lung cancer among high-risk patients [3], and recent studies showed the benefit of CT screening in community settings [5]. The wide adoption of lung cancer screening is expected to benefit millions of people [6]. However, millions of CT scan images obtained from patients constitute a heavy workload for radiologists [7]. In addition, interrater disagreement has been documented [8]. Previous studies suggested that computer-aided diagnostic systems could improve the detection of pulmonary nodules in CT examination [9-12]. To stimulate the development of machine learning models for automated CT diagnosis, the Kaggle Data Science Bowl provided labeled chest CT images from 1397 patients and awarded $1 million in prizes to the best algorithms for automated lung cancer diagnosis, which is the largest machine learning challenge on medical imaging to date. In response, 1972 teams worldwide have participated and 394 teams have completed all phases of the competition [13], making it the largest health care–related Kaggle contest [14]. This provides a unique opportunity to study the robustness of medical machine learning models and compare the performance of various strategies for processing and classifying chest CT images at scale.

Due to the improved performance of machine learning algorithms for radiology diagnosis, some developers have sought commercialization of their models. However, given the divergent software platforms, packages, and patches employed by different teams, their results were not easily reproducible. The difficulty in reusing the state-of-the-art models and reproducing the diagnostic performance markedly hindered further validation and applications.

To address this gap, we reimplemented, examined, and systematically compared the algorithms and software codes developed by the best-performing teams of the Kaggle Data Science Bowl. Specifically, we investigated all modules developed by the 10 award-winning teams, including their image preprocessing, segmentation, and classification algorithms. To ensure the reproducibility of results and the reusability of the developed modules, we generated a Docker image for each solution using the Docker Community Edition, a popular open-source software development platform that allows users to create self-contained systems with the desired version of software packages, patches, and environmental settings. According to Docker, there are over 6 million Dockerized applications, with 130 billion total downloads [15]. The Docker images are easily transferrable from one server to another, which ensures the reproducibility of scientific computing [16]. Our Dockerized modules will facilitate further development of computer-aided diagnostic algorithms for chest CT images and contribute to precision oncology.

## Methods

### Data and Classification Models

We obtained the low-dose chest CT datasets in Digital Imaging and Communications in Medicine format from the Kaggle Data Science Bowl website [13]. The dataset was acquired from patients with high risks for developing lung cancers. In this Kaggle challenge, a training set (n=1397) with ground truth labels (362 with lung cancer; 1035 without) and a public test set (n=198) without labels were provided to the participants. The ground truth label is 1 if the patient developed lung cancer within 1 year of the date the CT scan was performed and 0 otherwise. The diagnosis was confirmed by pathology evaluation as a part of the National Lung Screening Trial [3,17]. Once participants submitted the prediction results for the public test set, the Kaggle competition platform reported their models’ performance on the public leaderboard instantaneously. The final test set (n=506, ground truth labels were not disclosed to participants) was only available to participants after the model submission deadline, thus serving as an independent validation set that decided the final winners. The chest CT images in the training set, public test set, and final test set all came from multiple hospitals and had different qualities. In particular, the final test set contained more recent and higher quality data with thinner slice thickness than those in the two other sets [18].

To systematically compare the solutions developed by the award-winning teams, we acquired the source codes of the winning solutions and their documentation from the Kaggle news release after the conclusion of the competition. Per the rules of this Kaggle challenge, the source codes of these award-winning solutions were required to be released under open-source licenses approved by the Open Source Initiative [19] in order to facilitate free distribution and derivation of the solution codes [20]. The default license is the MIT license [20]. Under the open-source licenses approved by Open Source Initiative, the software can be freely used, modified, and shared [19].

### Comparison of the Approaches and Their Performance

We compared the workflows of the top 10 solutions by examining and rerunning their source codes. For each solution, we inspected all steps taken from inputting the CT images to outputting the prediction. We documented the versions of the software package and platform dependencies of each solution.

The Kaggle Data Science Bowl used the log-loss function to evaluate the performance of the models [13]. The log-loss function 
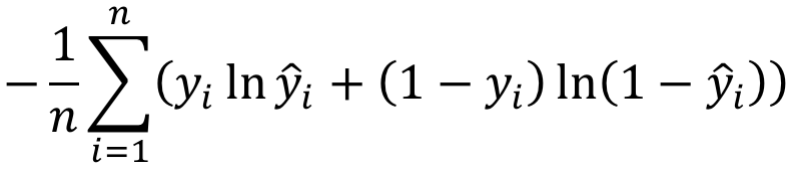
 where n is the number of patients in the test set, *y_i_* is 1 if patient i has lung cancer, 0 otherwise, and *ŷ_i_* is the predicted probability that patient i has lung cancer [13]. If the predicted outcome is set as 0.5 for all patients, the log-loss value would be ln (2) ≈ 0.69.

To investigate whether models with high performance in the public test set generalize to the images in the final test set, we computed the Spearman correlation coefficient of the log-loss in the two test sets. All analyses were conducted using R version 3.6 (R Foundation for Statistical Computing).

### Docker Image Generation for the Top Ten Solutions

We reproduced the results by recompiling the source codes and dependencies of each of the top ten solutions. Since the solutions used various platforms and different versions of custom software packages, many of which were not compatible with the most updated packages or mainstream release, we generated Docker images [16] to manage the software dependencies and patches required by each solution to enhance the reusability and reproducibility of the developed algorithms.

## Results

### Performance Comparison

**Figure 1:**
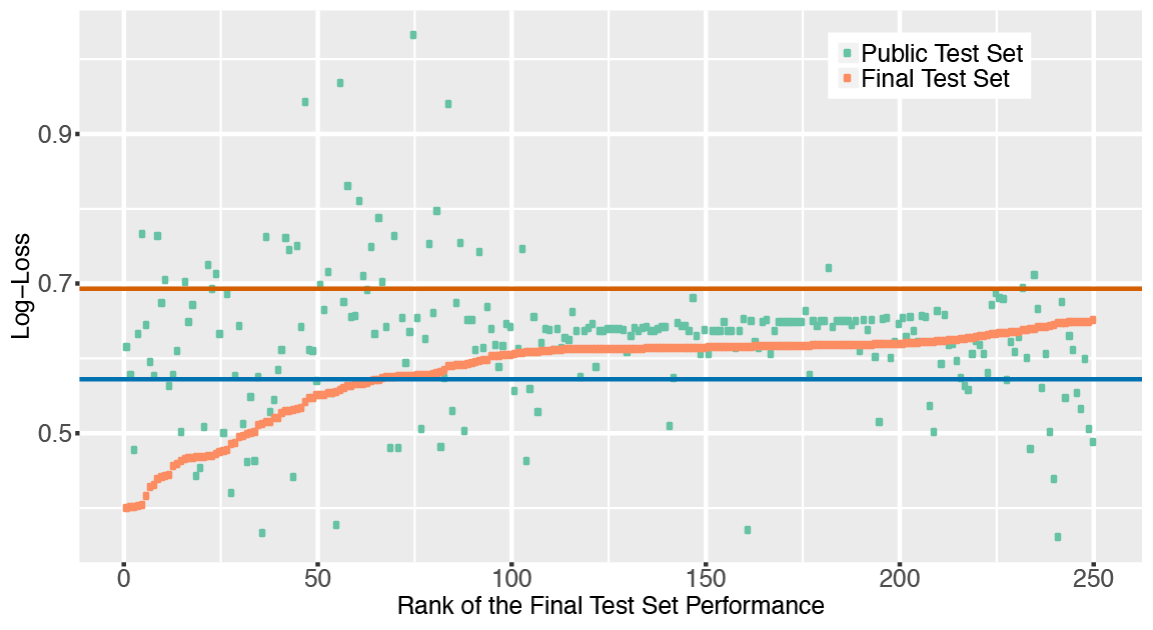
The log-loss score distribution of the top 250 teams in the Kaggle Data Science Bowl Competition. The log-loss scores of the public test set and the final test set of each team were plotted. The red horizontal line indicates the log-loss of outputting the cancer probability as 0.5 for each patient. The blue horizontal line shows the log-loss of outputting cancer probability of each patient as the prevalence of cancer (0.26) in the training set.

**Figure 2:**
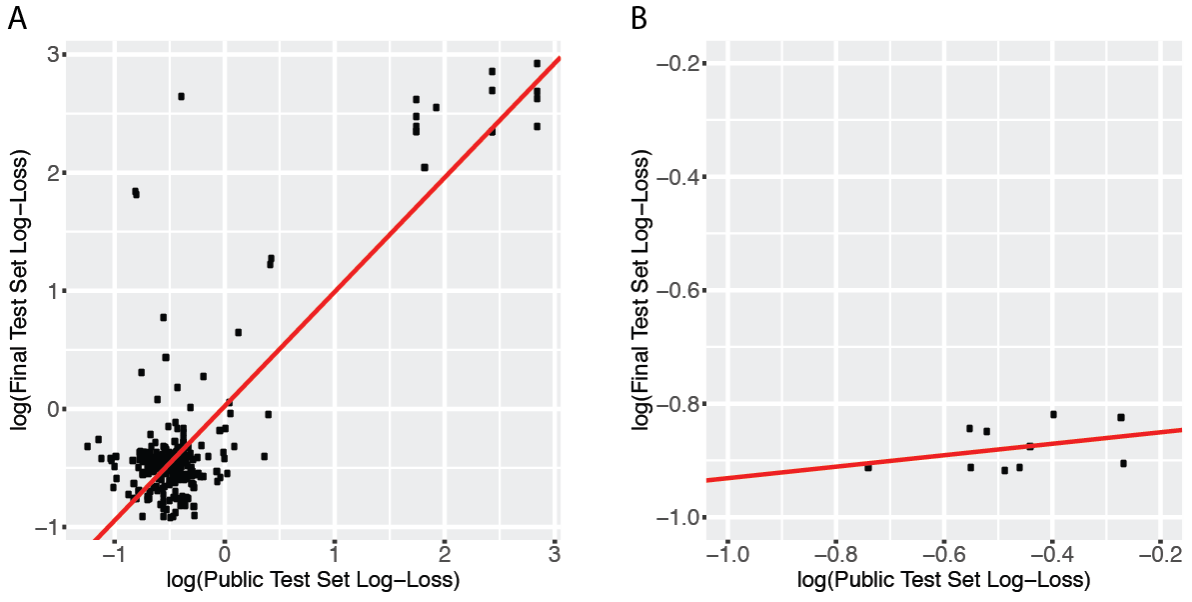
A weak to moderate correlation between the log-loss scores of the public test set and the scores of the final test set. The red regression line shows the relation between the log-loss scores of the public test set and those of the final test set using a linear regression model. (A) The log-transformed scores of all participants who finished both stages of the Kaggle Data Science Bowl Competition were plotted. The Spearman correlation coefficient of the performance in the two test sets is .23. (B) The log-transformed scores of the top 10 teams defined by the final test set performance. The Spearman correlation coefficient among the top 10 teams is .39.

Figure 1 summarizes the public and private test set scores of the top 250 teams that participated in the Kaggle Data Science Bowl. Results showed that the top 20 teams achieved a log-loss less than 0.5 in the final test set, and more than 80 submissions reached a log-loss less than 0.6 in the same set. However, these models had varying performances in the public test set. Surprisingly, 11 out of the top 50 teams had a public test set log-loss greater than 0.69, which was worse than blindly submitting “0.5” as the cancer probability for every patient. The correlation between the public test set scores and the final test set scores was weak among all teams that completed the contest (Spearman correlation coefficient = .23; Figure 2A). In the top 10 teams, the correlation is moderate (Spearman correlation coefficient = .39; Figure 2B).

### Data Workflow Comparison

**Figure 3:**
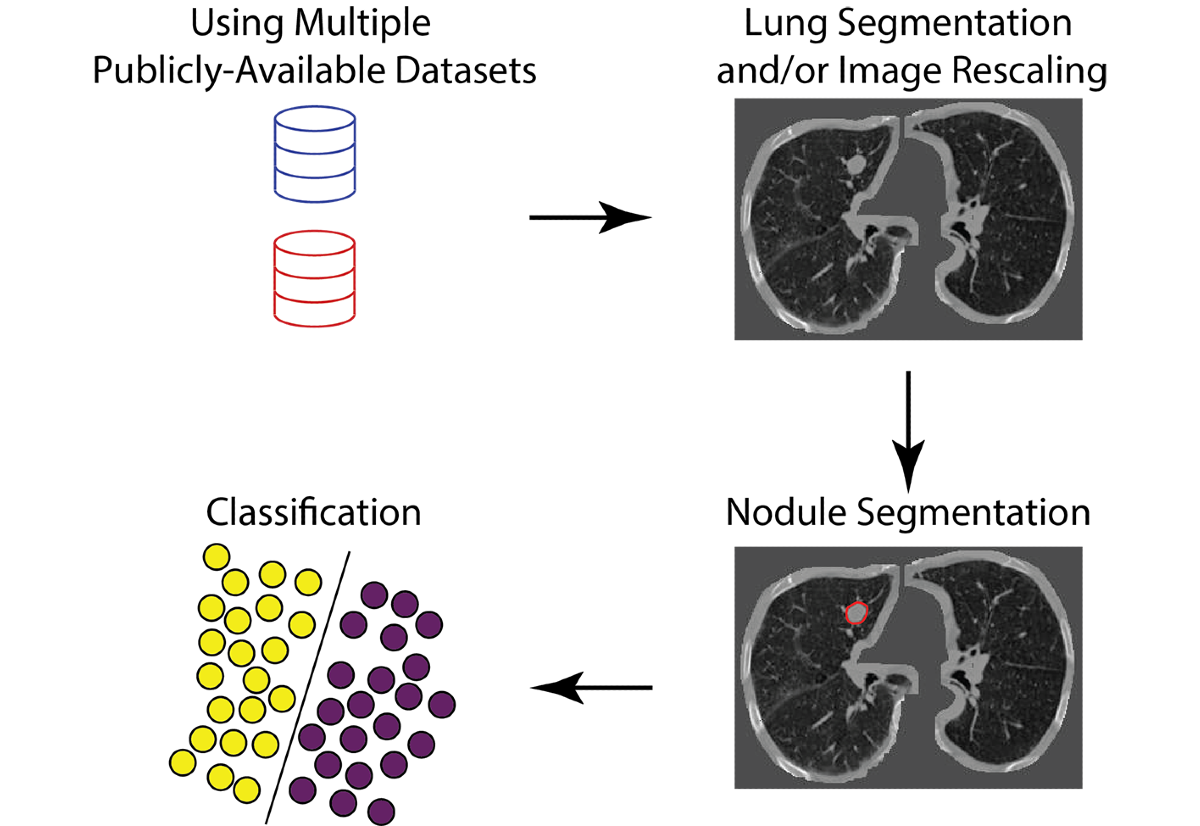
A model of the informatics workflow used by most teams. In addition to the Kaggle training set, most teams obtained additional publicly available datasets with annotations. Lung segmentation, image rescaling, and nodule segmentation modules were commonly used before classification.

Figure 3 summarizes the most frequently used strategy by the winning teams. Most solutions used additional publicly available datasets, generated lung segmentation, rescaled the voxels, and performed nodule segmentations before fitting the classification models. Table 1 compares the additional datasets, data preprocessing, segmentation, classification, implementation, and final test set scores of the top 10 solutions.

In addition to the training dataset provided by the Kaggle challenge, most teams used CT images and nodule annotations from other publicly available resources. Table 2 summarizes the sample size, availability of nodule locations, nodule segmentation, diagnoses, other characteristics of the Kaggle dataset, and additional datasets employed by the participants. Most of the top solutions used images and nodule segmentations from the Lung Nodule Analysis 2016 (LUNA16) challenge to develop their segmentation algorithms. LUNA16 is a closely related competition organized in 2016 with an aim to detect lung nodules in chest CT images [21,22]. Two teams also reported using the lung CT images, diagnostic annotations, and nodule location data from the International Society for Optics and Photonics (SPIE)–American Association of Physicists in Medicine (AAPM) Lung CT Challenge [23], but one of them did not incorporate this relatively small dataset (n=70) when building the final models. Only one of the top 10 teams did not use any additional datasets outside of the competition.

Frequently used image preprocessing steps include lung segmentation and voxel scaling. Voxel scaling ensures that the voxels of images from various CT scan protocols correspond to similar sizes of physical space. Variants of U-Net [24], VGGNet [25], and residual net (ResNet) [26] were commonly used as the nodule segmentation algorithms, and the nodule segmentation models trained on the LUNA16 dataset were often applied to the Data Science Bowl dataset.

After lung nodule segmentation, classification algorithms were employed to generate final cancer versus noncancer predictions. Most of the solutions leveraged existing ImageNet-based architecture and transfer learning [12,27]. All teams employed 2D or 3D convolutional neural networks (CNN). A few teams employed CNNs as feature extractors and used tree-based classifiers for this classification task.

**Table 1:** Comparisons of the top-performing solutions of the Kaggle Data Science Bowl.

Rank	Team name	Additional datasets used	Data preprocessing	Nodule segmentation	Classification algorithms	Implementation	Final test set score
1	Grt123	LUNA16^a^	Lung segmentation, intensity normalization	Variant of U-Net	Neural network with a max-pooling layer and two fully connected layers	Pytorch	0.39975
2	Julian de Wit and Daniel Hammack	LUNA16, LIDC^b^	Rescale to 1×1×1	C3D^c^, ResNet-like CNN^d^	C3D, ResNet-like CNN	Keras, Tensorflow, Theano	0.40117
3	Aidence	LUNA16	Rescale to 2.5×0.512×0.512 (for nodule detection) and 1.25×0.5×0.5 (for classification)	ResNet^e^	3D DenseNet^f^ multitask model (different loss functions depending on the input source)	Tensorflow	0.40127
4	qfpxfd	LUNA16, SPIE-AAPM^g^	Lung segmentation	Faster R-CNN^h^, with 3D CNN for false positive reduction	3D CNN inspired by VGGNet	Keras, Tensorflow, Caffe	0.40183
5	Pierre Fillard (Therapixel)	LUNA16	Rescale to 0.625×0.625×0.625, lung segmentation	3D CNN inspired by VGGNet	3D CNN inspired by VGGNet	Tensorflow	0.40409
6	MDai	None	Rescale to 1×1×1, normalize HU^i^	2D and 3D ResNet	3D ResNet + a Xgboost classifier incorporating CNN output, patient sex, # nodules, and other nodule features	Keras, Tensorflow, Xgboost	0.41629
7	DL Munich	LUNA16	Rescale to 1×1×1, lung segmentation	U-Net	2D and 3D residual neural network	Tensorflow	0.42751
8	Alex, Andre, Gilberto, and Shize	LUNA16	Rescale to 2×2×2	Variant of U-Net	CNN, tree-based classifiers (with better performance)	Keras, Theano, xgboost, extraTree	0.43019
9	Deep Breath	LUNA16, SPIE-AAPM^j^	Lung mask	Variant of SegNet	Inception-ResNet v2	Theano and Lasagne	0.43872
10	Owkin Team	LUNA16	Lung segmentation	U-Net, 3D VGGNet	Gradient boosting	Keras, Tensorflow, xgboost	0.44068

^a^LUNA16: Lung Nodule Analysis 2016.

^b^LIDC: Lung Image Database Consortium.

^c^C3D: convolutional 3D.

^d^ResNet-like CNN: residual net–like convolutional neural network.

^e^ResNet: residual net.

^f^DenseNet: dense convolutional network.

^g^SPIE-AAPM: International Society for Optics and Photonics–American Association of Physicists in Medicine Lung CT Challenge.

^h^R-CNN: region-based convolutional neural networks.

^i^HU: Hounsfield unit.

^j^Dataset has been evaluated but not used in building the final model.

**Table 2:** A summary of the chest computed tomography datasets employed by the participants.

Datasets	Number of CT^a^ scan series	Data originated from multiple sites	Availability of nodule locations	Availability of nodule segmentations	Availability of patients’ diagnoses (benign versus malignant)
Kaggle Data Science Bowl (this competition)	Training: 1397; public test set: 198; final test set: 506	Yes	No	No	Yes
Lung nodule analysis	888	Yes	Yes	Yes	Yes
SPIE-AAPM^b^ Lung CT Challenge	70	No	Yes	No	Yes
Lung Image Database Consortium	1398	Yes	Yes	Yes	Yes

^a^CT: computed tomography.

^b^SPIE-AAPM: International Society for Optics and Photonics–American Association of Physicists in Medicine.

### Comparison of the Implementation Platforms and Software Dependencies

**Figure 4:**
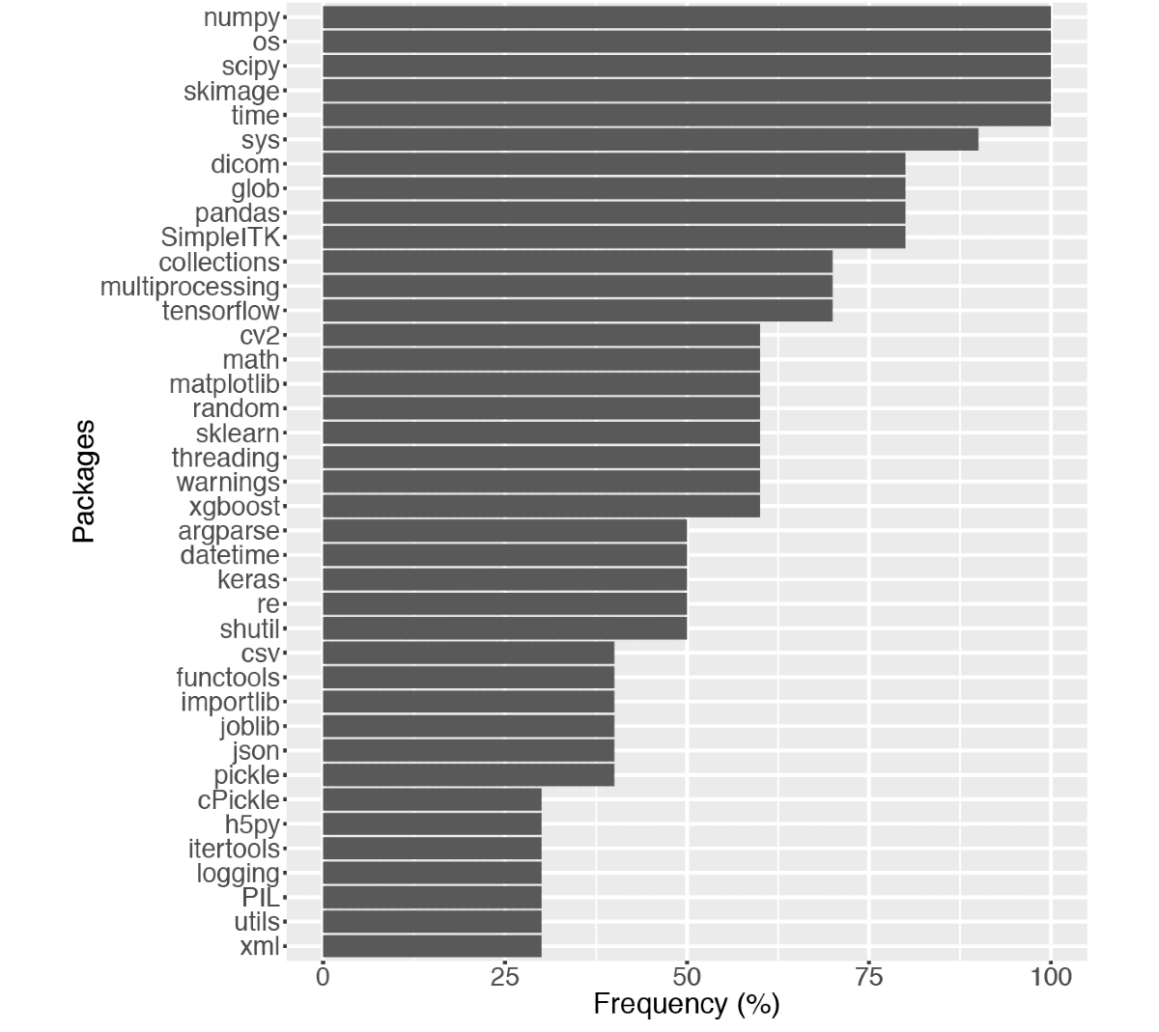
The most widely used dependencies by the top 10 teams. The packages are ordered by their prevalence among the top teams. For simplicity, dependencies used by only one team are omitted from the figure.

Most of the winning teams developed their modules with Keras and Tensorflow. Only one team used Pytorch (the top-performing team), Caffe, or Lasagne. All of the top 10 teams employed a number of python packages for scientific computing and image processing, including NumPy, SciPy, and Scikit-image (skimage). A summary of package dependencies is shown in Figure 4. This reflected the popularity of the tools for processing chest CT images, building neural networks, and scientific computing among the top contestants of this contest.

### Docker Images of the Top Solutions

To facilitate reusing the code developed by the top teams, we generated a Docker image for each of the available solutions. Our developed Docker images are redistributed under the open-source licenses chosen by the original developers [28]. Detailed instructions on accessing the Docker images can be found on GitHub [29].

## Discussion

### Principal Findings

This is the first study that systematically compared the algorithms and implementations of award-winning pulmonary nodule classifiers. Results showed that the majority of the best-performing solutions used additional datasets to train the pulmonary nodule segmentation models. The top solutions used different data preprocessing, segmentation, and classification algorithms. Nonetheless, they only differ slightly in their final test set scores.

The most commonly used data preprocessing steps were lung segmentation and voxel scaling [30]. For nodule classification, many solutions used CNNs. However, 2 of the top 10 teams employed tree-based methods for cancer versus noncancer classification. Tree-based approaches require a predefined set of image features, whereas CNNs allow data to refine the definition of features [31]. Given sufficient sample size, CNNs outperformed tree-based methods in many image-related tasks [12,32], whereas tree-based methods could reach satisfactory performance when the sample size was small, and they provided better model interpretability [33-35]. Since the conclusion of the contest, additional works on machine learning for CT evaluation have been published [36-40]. Nonetheless, these works reported similar strategies for data processing and classification overall.

To enhance the reproducibility of the developed modules, we generated a Docker image for each of the award-winning solutions. The Docker images contain all software dependencies and patches required by the source codes and are portable to various computing environments [16], which will expedite the application and improvement of the state-of-the-art CT analytical modules implemented by the contest winners.

### Limitations

Since it was difficult to compile and release a large deidentified chest CT dataset to the public, the public test set only contains images from 198 patients. Leveraging the 5-digit precision of the log-loss value shown on the leaderboard, one participant implemented and shared a method for identifying all ground truth labels in the public test set during the competition [41]. Several participants successfully replicated this approach and got perfect scores on the public leaderboard. Thus, solutions with very low log-loss in the public test set may result from information leakage. Interestingly, among the top-10 models defined by the final test set, 2 performed worse than random guessing in the public test set, which raised concerns on their generalizability [42].

There are several approaches future contest organizers can take to ensure the generalizability of the developed models. First, a multistage competition can filter out the overfitted models using the first private test set and only allow reasonable models to advance to the final evaluation. In addition, organizers can discourage leaderboard probing by only showing the performance of a random subset of the public test data or limiting the number of submissions allowed per day. Finally, curating a larger test set can better evaluate the true model performance and reduce random variability [43]. If data deidentification is difficult, requiring contestants to submit their models to a secure computing environment rather than distributing the test data to the participants can minimize the risk of leaking identifiable medical information.

### Conclusion

In summary, we compared, reproduced, and Dockerized state-of-the-art pulmonary nodule segmentation and classification modules. Results showed that many transfer learning approaches achieved reasonable accuracy in diagnosing chest CT images. Future works on additional data collections and validation will further enhance the generalizability of the current methods.

## Abbreviations

AAPM: American Association of Physicists in Medicine
CNN: convolutional neural network
CT: computed tomography
LUNA16: Lung Nodule Analysis 2016
ResNet: residual net
skimage: Scikit-image
SPIE: International Society for Optics and Photonics
